# Human NK Cell Subset Functions Are Differentially Affected by Adipokines

**DOI:** 10.1371/journal.pone.0075703

**Published:** 2013-09-30

**Authors:** Lena Huebner, Stefan Engeli, Christiane D. Wrann, Lilia Goudeva, Tobias Laue, Heike Kielstein

**Affiliations:** 1 Institute for Functional and Applied Anatomy, Hannover Medical School, Hannover, Germany; 2 Department of General-, Visceral-, Vascular and Thoracic Surgery, Charité, University Medicine Berlin, Campus Mitte, Berlin, Germany; 3 Institute for Clinical Pharmacology, Hannover Medical School, Hannover, Germany; 4 Division of Endocrinology, Beth Israel Deaconess Medical Center, Harvard Medical School, Boston, Massachusetts, United States of America; 5 Institute for Transfusion Medicine, Hannover Medical School, Hannover, Germany; 6 Department of Anatomy and Cell Biology, Martin Luther University Halle-Wittenberg, Faculty of Medicine, Halle (Saale), Germany; University of Sydney, Australia

## Abstract

**Background:**

Obesity is a risk factor for various types of infectious diseases and cancer. The increase in adipose tissue causes alterations in both adipogenesis and the production of adipocyte-secreted proteins (adipokines). Since natural killer (NK) cells are the host’s primary defense against virus-infected and tumor cells, we investigated how adipocyte-conditioned medium (ACM) affects functions of two distinct human NK cell subsets.

**Methods:**

Isolated human peripheral blood mononuclear cells (PBMCs) were cultured with various concentrations of human and murine ACM harvested on two different days during adipogenesis and analyzed by fluorescent-activated cell sorting (FACS).

**Results:**

FACS analyses showed that the expression of tumor necrosis factor-related apoptosis-inducing ligand (TRAIL), granzyme A (GzmA) and interferon (IFN)-γ in NK cells was regulated in a subset-specific manner. ACM treatment altered IFN-γ expression in CD56^dim^ NK cells. The production of GzmA in CD56^bright^ NK cells was differentially affected by the distinct adipokine compositions harvested at different states of adipogenesis. Comparison of the treatment with either human or murine ACM revealed that adipokine-induced effects on NK cell expression of the leptin receptor (Ob-R), TRAIL and IFN-γ were species-specific.

**Conclusion:**

Considering the growing prevalence of obesity and the various disorders related to it, the present study provides further insights into the roles human NK cell subsets play in the obesity-associated state of chronic low-grade inflammation.

## Introduction

Obesity, defined as a body-mass-index (BMI) ≥30 kg/m^2^, is one of today’s leading health threats in most industrialized countries [1]. Data from the National Health and Nutrition Examination Survey (NHANES) 2005–2006 show that more than 34% of the US population aged 20 years and older were obese [2]. Excess body weight is associated with the manifestation of several disorders, such as type 2 diabetes, cardiovascular disease, hypercholesterolaemia and hypertension [3]. Furthermore, obesity increases the susceptibility to infections and the risk to develop multiple types of cancer (e.g. colon and postmenopausal breast cancer) [4], [5]. These findings led to the suggestion of obesity being an immunodeficient state [6].

White adipose tissue, which is highly expanded in obesity, acts as an endocrine organ that actively participates in physiological and pathological processes, including immunity and inflammation [7]. Among the secreted adipokines with relevance for immunological processes are tumor necrosis factor (TNF)-α, interleukin (IL)-6, leptin, adiponectin, resistin and visfatin [8], [9]. Adiponectin’s influence on immune functions seems to be mainly anti-inflammatory as it suppresses the production and secretion of the pro-inflammatory cytokines TNF-α, IL-6 and interferon (IFN)-γ by LPS-activated macrophages, while the anti-inflammatory cytokines IL-10 and IL-1 receptor antagonist are induced in adiponectin-treated monocytes, macrophages and dendritic cells [10], [11]. Leptin, resistin and visfatin are considered pro-inflammatory adipokines. They induce pro-inflammatory cytokines [12], [13] and their plasma concentrations are elevated during inflammatory conditions [14]. Plasma leptin is elevated during obesity [15], and cells of both the innate and adaptive immunity are influenced by leptin [16]. Leptin-deficient children have been reported to suffer more often from infections than their healthy siblings and to show impaired numbers and function of T cells [17], thus implicating a role for leptin as a link between nutritional and immunological status of the organism. Previous *in vitro* and *in vivo* studies conducted by our group demonstrated an impaired leptin-dependent signal transduction in natural killer (NK) cells in diet-induced obesity (DIO) which could be ameliorated by transfer of NK cells to a normal-weight metabolic environment [18], [19].

NK cells are an integral component of the innate immune system. They produce cytokines (e.g. IFN-γ) that stimulate other immune cells and they destroy infected or transformed cells [20]. NK cells express a variety of activating and inhibitory receptors which determine their specificity for divergent targets [21]. Adiponectin is a negative regulator of IL-2-induced NK cell cytotoxicity and of IFN-γ production by NK cells [22]. The effects of a composition of adipocyte-derived factors on NK cell immunity, however, remain unclear. NK cells are the central active component of the host’s immune system in the early phase of cancer development and metastasis. Since adipokines have been suggested to represent a possible link between obesity and cancer [23], a detailed investigation on the influence of adipokines on human NK cell functions is warranted. The aim of the present study was to show how adipocyte-derived proteins may contribute to a compromised immune response during obesity by systematically analyzing effects of human SGBS and murine 3T3-L1 adipocyte-conditioned medium (ACM) on human NK cell subsets.

The SGBS cell strain was established from a subcutaneous adipose tissue sample of a male infant with Simpson-Golabi-Behmel (SGBS) syndrome. It is utilized as an *in vitro* model for human adipocyte differentiation and shows a protein secretion pattern comparable to differentiating human preadipocytes in primary culture [24].

## Materials and Methods

### Subjects

Leukocyte filters were obtained shortly after processing of blood donations from healthy donors. Since the study is an investigation of anonymous blood probes no written or verbal consent was provided. For this study a formal written waiver (issued by the head of the ethics committee Professor Tröger) was obtained from the ethics committee. All research was performed at the Hannover Medical School.

**Table 1:** Lymphocyte numbers after 24 hours of stimulation with ACM (adipocte-conditioned medium).

	Medium	SEM	0.1% ACM	SEM	1% ACM	SEM	10% ACM	SEM
NK cells	13.1	2.2	14.1	3.6	14.3	3.7	14.1	3.6
CD56^dim^ NK cells	93.2	1.0	93.3	1.4	92.9	1.4	92.7	1.8
CD56^bright^ NK cells	6.8	1.0	6.7	1.5	7.1	1.4	7.3	1.8
T cells	59.9	3.0	60.8	2.9	60.9	3.1	61.2	2.8
NKT cells	3.4	1.0	2.4	0.7	2.3	0.7	2.4	0.7

Human peripheral blood mononuclear cells (PBMCs) were isolated from six leukocyte filters and cultured for 24 hours with R10-medium conditioned with 0.1%, 1% or 10% of SGBS ACM. PBMCs incubated for 24 hours with medium only served as controls. A detailed description of the incubation procedure can be found in the materials and methods section. NK cells, T cells and NKT cells are shown as percent of lymphocytes. CD56^dim^ and CD56^bright^ NK cells are shown as percent of all NK cells.

For the stimulation with each of the different ACM, six leukocyte preparations were made out of six filters. The samples were categorized according to the BMI of the blood donors into two groups: three normal-weight (nw, BMI <25 kg/m^2^) and three overweight (ow, BMI ≥25 kg/m^2^). In the nw group used for stimulation with SGBS ACM harvested on day 7 the BMI range was 20–24 with a mean age of 52 years and in the corresponding ow group the BMI range was 25–29 with a mean age of 55 years. In the nw group used for stimulation with SGBS ACM from day 11 the BMI range was 18–24 with a mean age of 43 years and the corresponding ow group had a BMI range of 25–28 with a mean age of 44 years. The PBMCs stimulated with 3T3-L1 ACM harvested on day 7 were derived from three nw (BMI range 20–24, mean age 43 years) and three ow (BMI range 25–27, mean age 56 years) blood donors to match the samples stimulated with the SGBS ACM from day 7.

### PBMC Isolation and Culture

PBMCs were isolated from leukocyte filters used for the preparation of erythrocyte concentrates. Filters were obtained from the Institute for Transfusion Medicine, Hannover Medical School, shortly after blood passage and flushed with phosphate-buffered saline (PBS) (Dulbecco, Pasching, Austria) in the opposite direction of original blood flow. The extracted leukocytes were separated by density gradient centrifugation for 20 minutes at 1000×*g* and 4°C using biocoll separating solution (Biochrom AG, Berlin, Germany). PBMCs from the interphase were washed with PBS for 10 minutes at 1000×*g* and 4°C and for another 10 minutes at 400×*g* and 4°C, resuspended in RPMI-1640 medium supplemented with 10% fetal calf serum (FCS), 100 U/ml penicillin, 100 µg/ml streptomycin, 2 mM L-glutamine and 1 mM sodium pyruvate (Biochrom AG) (R10-medium) and adjusted to 2×10^6^ cells/ml. The PBMCs were cultured in 96-well round-bottom cell culture plates (Greiner Bio-One GmbH, Solingen, Germany) (5×10^5^ cells/well) in R10-medium supplemented with 100 iu/ml Interleukin-2 (IL-2) (Eurocetus, Amsterdam, the Netherlands) at 37°C in a humidified atmosphere with 5% CO_2_.

### SGBS Adipocyte-conditioned Medium

SGBS preadipocytes were seeded at a density of 4000 cells/cm^2^ and grew to 80% confluence in adipocyte growth medium (DMEM medium plus 10% FBS, Invitrogen, Karlsruhe, Germany, supplemented with 300 µM biotin, 150 µM panthothenat, 100 U/ml penicillin and 100 µg/ml streptomycin, all from Sigma-Aldrich, Seelze, Germany). To initiate adipogenic differentiation, cells were washed with PBS and incubated for three days with adipogenesis medium 1 (adipocyte growth medium as above without FBS plus 20 µM insulin, 0.2 nM triiodothyronine, 0.01 mg/ml human transferrin, 500 µM IBMX, 0.25 nM dexamethasone and 10 µM pioglitazone, all from Sigma-Aldrich, Seelze, Germany). At day four, medium was changed to adipogenesis medium 2 (adipocyte growth medium as above without FBS plus 20 µM insulin, 0.2 nM triiodothyronine and 0.01 mg/ml human transferrin). To obtain adipocyte-conditioned medium, we collected the supernatants at days 7 and 11, centrifuged the supernatants at 5000×*g* for 10 minutes and collected the ACM without transferring any cellular debris. ACM was stored at −80°C and sterile-filtered before use.

### 3T3-L1 Adipocyte-conditioned Medium

3T3-L1 cells were grown in DMEM high glucose (Gibco®, Invitrogen Carlsbad, CA) with 10% bovine calf serum (Hyclone, Thermo Fisher Scientific, Waltham, MA). Two days after confluence differentiation was induced with 800 nM insulin, 1 µM dexamethason and 0.5 mM 3-isobutyl-1-methylxanthine in DMEM high glucose with 10% FBS (Atlas Biological, Fort Collin, CO) for three days, followed by two days with 800 nM insulin and 1 µM dexamethason. ACM was collected at days 7 and 11 after induction, stored at −80°C and sterile-filtered before use.

### Cell Stimulation

PBMCs were stimulated directly after isolation or after one or two days in culture with either SGBS or 3T3-L1 ACM harvested on day 7 or 11 after the induction of adipogenic differentiation (as described above). Portions of 1% of the PBMC culture medium were substituted with either SGBS or 3T3-L1 ACM harvested on one of the respective days and 100 U/ml IL-2 (Eurocetus) was supplemented. In cases where intracellular staining for cytokines was performed, brefeldin A (2 µg/ml; Sigma-Aldrich, Seelze, Germany) was added 1 h after the beginning of cell stimulation to inhibit cytokine secretion. Cells were cultivated with the conditioned medium for 24 hours at 37°C in a humidified atmosphere with 5% CO_2_. A well of cells treated with R10-medium plus IL- served as control.

### Cytospot Preparation and Immunohistology

**Figure 1:**
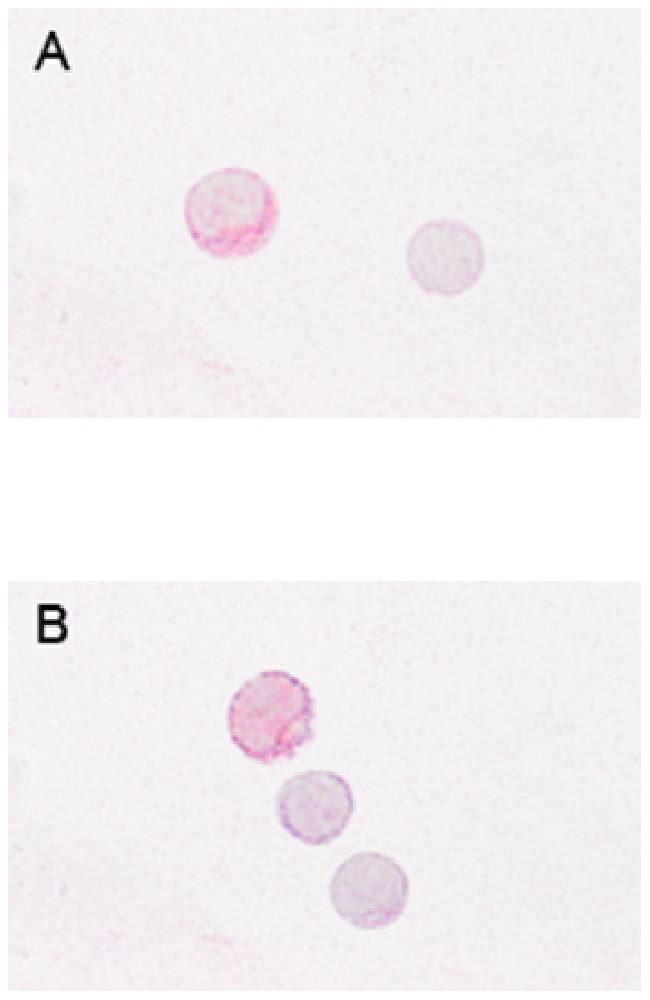
Cytospots prepared with human peripheral blood mononuclear cells (PBMCs) isolated from leukocyte filters or heparinized blood showed no morphological differences in the lymphocytes resulting from the preparation techniques. A) PBMCs isolated from a leukocyte filter. The left cell displays an NK cell expressing CD56 (red) and the leptin receptor (Ob-R, blue). The hematoxylin stained lymphocyte (right) is negative for CD56 and Ob-R. B) The representative cytospot prepared with PBMCs isolated from heparinized blood shows a CD56/Ob-R double-positive NK cell (top), a CD56-positive lymphocyte (middle) and a double-negative lymphocyte counterstained in hematoxylin.

PBMCs were isolated from either leukocyte filters or from heparinized blood. The heparinized blood samples were also subjected to density gradient centrifugation and PBMCs extracted from the interphase as described above. Afterwards, the cells were maintained in R10-medium. Cells were washed with PBS for 10 minutes at 400×*g* and 4°C, adjusted to 750×10^3^/ml in autoMACS™ Rinsing Solution (Miltenyi Biotec GmbH, Bergisch Gladbach, Germany) and 100 µl were applied per spot on SuperFrost®Plus microscope slides (Menzel-Gläser, Braunschweig, Germany) and centrifuged for 5 minutes at 400×*g* in a Shandon Cytospin3 centrifuge (Life Sciences International Ltd, Cheshire, England). The cytospots were air-dried for 2 hours and preserved at −20°C until staining. The unthawed preparations were immersion fixed with methanol-acetone (1∶1) for 10 minutes, rehydrated with tris-phosphate buffered saline (TBS)-Tween (1∶5; Serva, Boehringer Ingelheim, Germany) and afterwards incubated with the primary mouse anti human antibody directed against the leptin receptor Ob-R (sc-8391; Santa Cruz Biotechnology, Inc., Heidelberg, Germany; dilution 1∶100 in PBS 1 M, 1% BSA (Serva, Heidelberg, Germany), 0.1% Na-azide (Merck, Darmstadt, Germany) (measuring buffer)) overnight at 4°C in a humid chamber. After rinsing in TBS-Tween, cytospots were incubated with rabbit anti mouse immunoglobulins (Z259; 1∶50; DakoCytomation, Glostrup, Denmark) supplemented with 5% Pentaglobin ([∼76%IgG, ∼12% IgM, ∼12% IgA], Biotest Pharma GmbH, Dreieich, Germany) to prevent non-specific binding via Fc receptors. To visualize the binding, the APAAP-complex (monoclonal mouse antibodies D651; 1∶50 in TBS for 30 minutes; DakoCytomation) was utilized, followed by the repetition of the last two steps for 15 minutes. The cytospots were stained with Fast Blue (Fast Blue BB Salt, Sigma-Aldrich, St. Louis, USA) for 25 minutes, washed with TBS-Tween and incubated with a second primary mouse anti human antibody directed against CD56 (clone NCAM16.2;BD Biosciences, Heidelberg, Germany; dilution 1∶7500 in measuring buffer) for 30 minutes. After rinsing in TBS-Tween, APAAP-labeling was repeated as described above and cytospots were stained with Fast Red (Fast Red TR Salt, Sigma-Aldrich) for 25 minutes, counterstained with hematoxylin (1∶5 in PBS; Mayer’s hemalaun solution, Merck, Darmstadt, Germany) for 30 seconds and mounted in glycergel (DakoCytomation). Method specificity was tested by omission of the primary antibodies. Light microscopy was performed with an Axiophot microscope (Carl Zeiss) to qualitatively analyze possible morphological alterations in the NK cells isolated from leukocyte filters as compared to cells extracted from heparinized blood. As depicted in Figures 1A and 1B no morphological difference resulted from the cell preparation technique.

### Phenotypic Analyses

For phenotypic analyses after 24 hours of stimulation (as described above), PBMCs were stained with the directly labelled monoclonal mouse-anti human antibodies CD3 conjugated with phycoerythrin (PE)-Cy7 (CD3-PE-Cy7) (clone SK7, 1∶50), CD56 conjugated with allophycocyanin (CD56-APC) (clone NCAM16.2, 1∶100), CD253-PE (TRAIL; clone RIK-2, 1∶20) (BD Biosciences, San Diego, CA) and anti-hLeptin R (Ob-R) conjugated with carboxyfluorescein (anti-hLeptin R-CFS) (clone # 52263, 1∶20) (R&D Systems, Wiesbaden, Germany). PBMCs (5×10^5^ cells/100 µl) were incubated in 96 well-round bottom plates with the above mentioned antibodies for 20 minutes at 4°C, washed twice with measuring buffer and analyzed by flow cytometry using a FACSCanto (BD Biosciences, San Jose, CA) with FACS Diva software v5.0.3. A well with cells stained with the above mentioned antibodies except for anti-hLeptin R-CFS served as control for the measurements of Leptin R.

### Intracellular Staining

After 24 hours of cell culture and another 24 hours of stimulation (as described above) the expression of intracellular cytokines by NK cells was analyzed using a FACSCanto cytometer. Prior to intracellular labelling cell surface staining was performed. PBMCs (5×10^5^ cells/100 µl in 96-well round-bottom plates) were stained with CD3-PE (1∶250) and CD56-APC (1∶100). After 20 minutes at 4°C, cells were washed twice with measuring buffer and incubated with PBS supplemented with 4% paraformaldehyde (Merck, Darmstadt) in the dark for 10 minutes at room temperature. Cells were washed once with measuring buffer and once with saponin buffer (aqua dest. supplemented with 0.1% saponin and 0.01 M HEPES), then resuspended in saponin buffer and stained with the directly labelled mononuclear mouse anti-human antibodies granzyme A (GzmA) conjugated with fluorescein isothiocyanate (GzmA-FITC) (clone CB9, 1∶100) and IFN-γ-PE-Cy7 (clone 4 S.B3, 1∶100) (both BD Biosciences). To prevent non-specific binding via Fc receptors, each well was supplemented with 5 µl Pentaglobin. The data from flow cytometric analyses were processed with FACS Diva software v5.0.3. A well with cells stained with the above mentioned antibodies except for IFN-γ-PE-Cy7 served as control for the measurements of IFN-γ expression by NK cells.

### Conjugate-forming Assay

After two days of culture and 24 hours of stimulation (as described above), cell surface staining of the PBMCs (5×10^5^ cells/100 µl) was performed in 96-well round-bottom plates by adding CD3-PE (1∶250) and CD56-APC (1∶100) and incubating for 20 minutes at 4°C. After two washes, each well was supplemented with 8×10^4^ cells of the K562 erythroleukemia line (which were maintained in suspension culture flasks at 37°C in a humidified atmosphere with 5% CO_2_) and replenished with R10-medium up to a total volume of 240 µl. The cells were lightly centrifuged at 100×*g* for 3 minutes at 4°C and incubated for 20 minutes at 37°C, 5% CO_2_ and 85% RH. Cells were carefully resuspended and transferred into FACS tubes using pipet tips with expanded apertures. After gently mixing the cells, conjugate formation was analyzed using a FACSCanto by gating on PBMC and K562 cells, excluding CD3^+^ T cells.

K562 cells display a green autofluorescence which was detected in the FL1 channel. By gating on clusters fluorescing green (autofluorescence) and blue (CD56-APC) simultaneously, NK cells bound to K562 target cells could be identified.

### Statistics

Data are expressed as means+SEM. Significance was assigned when p<0.05. Data sets were analyzed using one-way ANOVA with Tukey multiple comparison test for post hoc analysis. The software used was with StatView Version 5.0, SAS Institute Inc., Cary, NC.

## Results

Human peripheral blood mononuclear cells (PBMCs) were cultured for 24 hours with R10-medium conditioned with 1% of SGBS ACM harvested on day 7 (d7) or day 11 (d11) after induction of adipogenesis. For each of the two different ACM treatments six different leukocyte preparations were analyzed. PBMCs incubated for 24 hours with medium only served as controls. Here we mainly show results after stimulation with ACM harvested on day 7 when stimulation with the two different ACM lead to comparable findings. Incubation with medium only and incubation with 50% R10-medium and 50% fresh adipogenesis medium 2 (that has not been used to culture SGBS cells and therefore does not contain adipokines) led to comparable results (data not shown). All significant differences shown after ACM stimulation versus incubation with medium only were also seen after comparison of ACM stimulation with incubation with 50% R10-medium and 50% adipogenesis medium 2. Therefore we could exclude that effects after ACM stimulation were influenced by supplements to the adipogenesis medium such as e.g. triiodothyronine.

### Lymphocyte Numbers are not Altered After 24 Hours of ACM Stimulation

After 24 hours of SGBS ACM stimulation, cell numbers were not significantly changed as compared to the medium control. Independent of the treatment conditions the proportions of NK cells, T cells and NKT cells as percentage of lymphocytes remained comparable, as did numbers of CD56^dim^ and CD56^bright^ NK cells as percentage of all NK cells (Table1).

### Expression of the Leptin Receptor is Comparable in Both Human NK Cell Subsets Before and After ACM Stimulation

**Figure 2:**
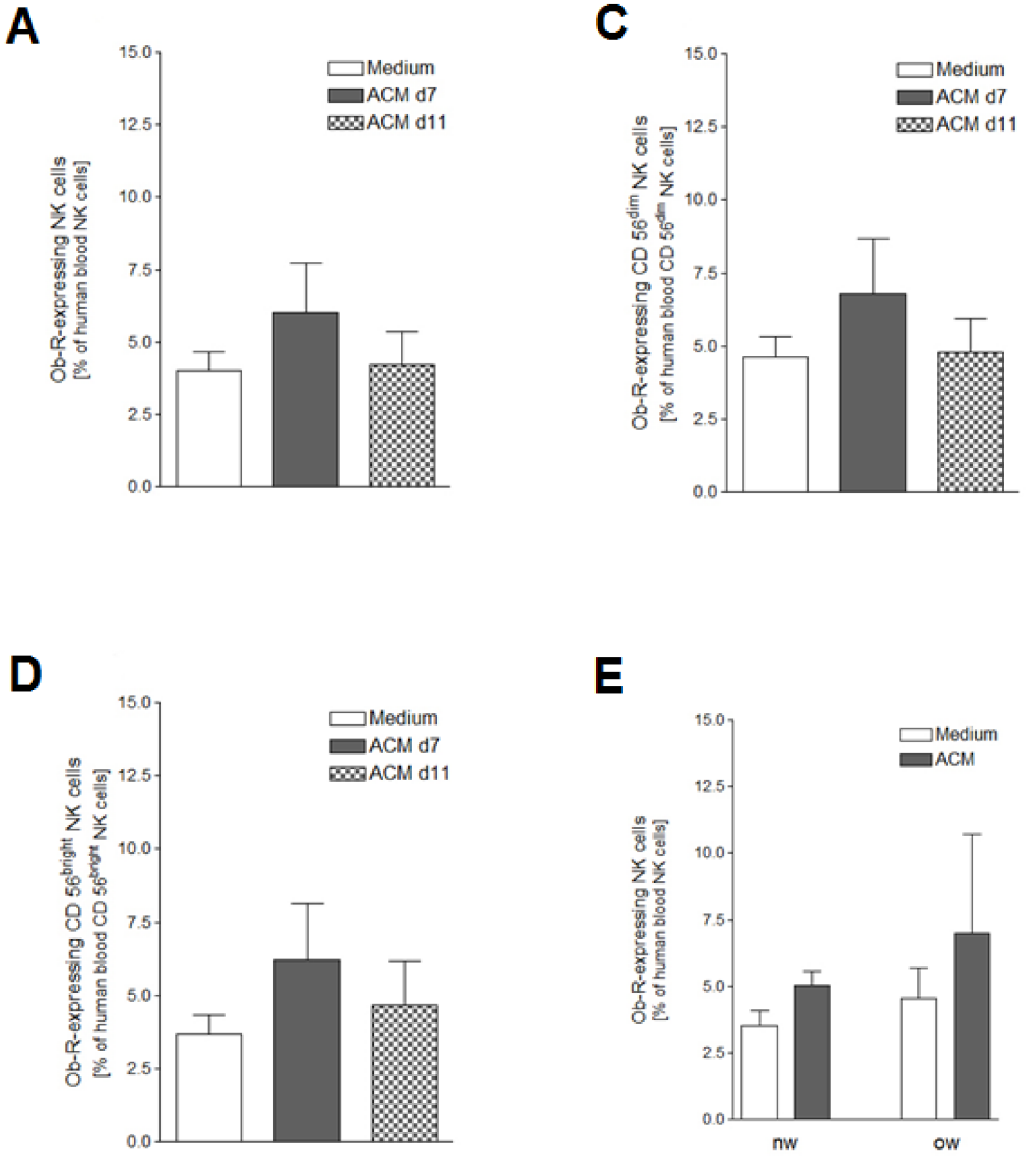
Ob-R (leptin receptor) expression after ACM stimulation. Human peripheral blood mononuclear cells (PBMCs) isolated from six leukocyte filters in each case were cultured for 24 hours with R10-medium conditioned with 1% of SGBS ACM harvested on day 7 or day 11 after induction of adipogenic differentiation. PBMCs incubated for 24 hours with medium only served as controls. A detailed description of the incubation procedure can be found in the materials and methods section. A) Ob-R-expressing human blood NK cells as percentage of human blood NK cells; B) Ob-R-expressing human blood CD56^dim^ NK cells as percentage of human blood CD56^dim^ NK cells; C) Ob-R-expressing human blood CD56^bright^ NK cells as percentage of human blood CD56^bright^ NK cells. D) Ob-R-expressing human blood NK cells as percentage of human blood NK cells from lean (nw, BMI <25, n = 3) and overweight (ow, BMI ≥25, n = 3) humans after 24 hours of stimulation with 1% SGBS ACM harvested on day 7 after induction of adipogenesis, controls were incubated for 24 hours with medium only. All data represent means+SEM.

In a previous study we could show that blood NK cells from DIO rats show a significant up-regulation of the leptin receptor isoform Ob-Rb in comparison to lean littermates [19]. Thus, we investigated the effect of a 24 hours stimulation with human SGBS ACM on the Ob-R expression of human peripheral blood NK cells. About 4–8% of the NK cells expressed Ob-R (Fig.2A). The two NK cell subgroups showed a comparable Ob-R expression pattern (Fig.2B, C). Comparison of the Ob-R expression patterns of NK cells from overweight (n = 3) and normal-weight subjects (n = 3) showed a tendency towards higher numbers of Ob-R-positive NK cells in the blood of overweight individuals (Fig.2D).

### TRAIL Expression is Higher in CD56^bright^ than in CD56^dim^ NK Cells

**Figure 3:**
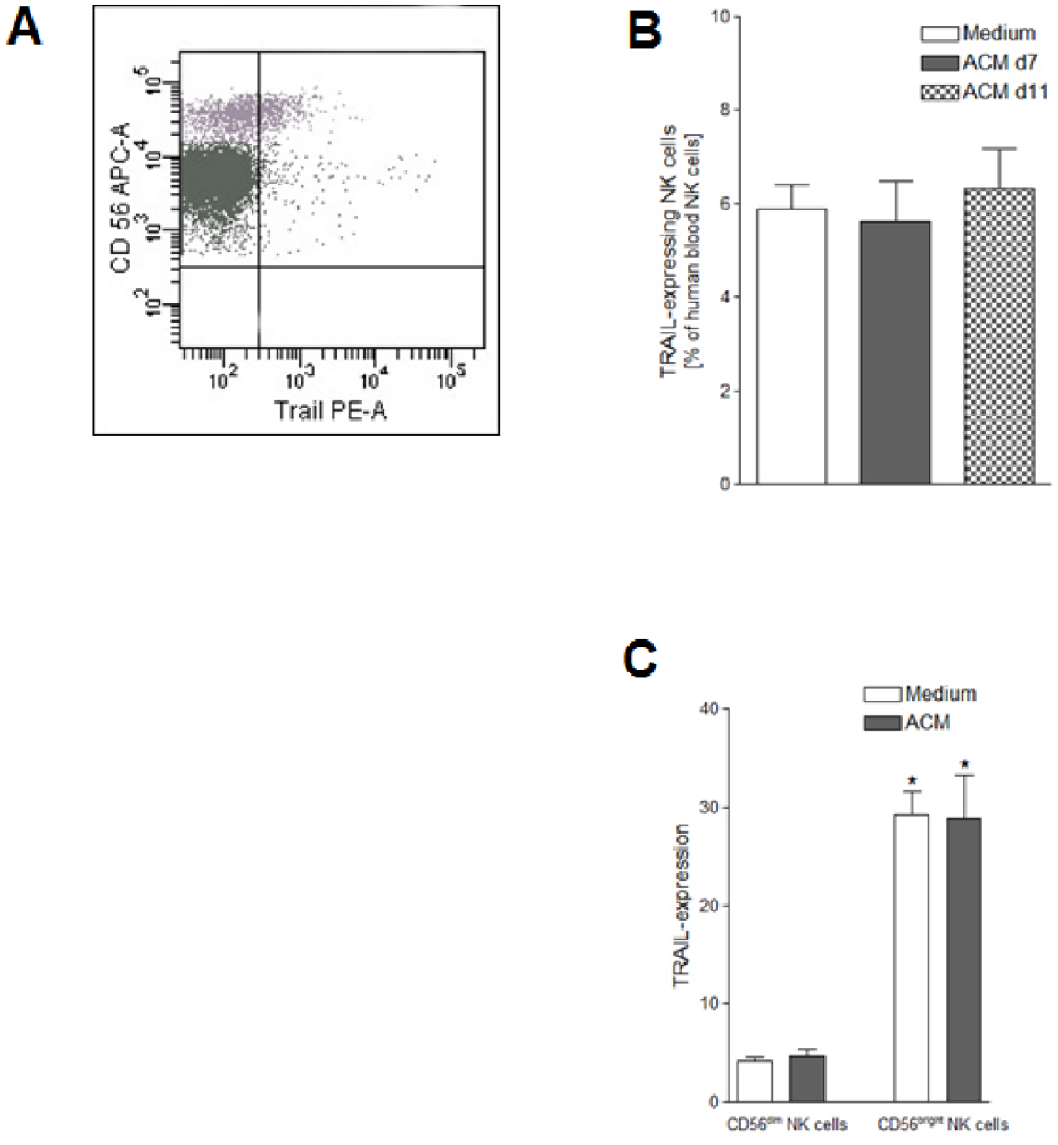
TRAIL expression after ACM stimulation. Human peripheral blood mononuclear cells (PBMCs) were isolated from six leukocyte filters in each case and cultured for 24 hours with R10-medium conditioned with 1% of SGBS ACM harvested on day 7 or day 11 after induction of adipogenesis. PBMCs incubated for 24 hours with medium only served as controls. A detailed description of the incubation procedure can be found in the materials and methods section. A) TRAIL-expressing human blood NK cells as percentage of human blood NK cells; B) TRAIL-expressing human blood CD56^dim^ and CD56^bright^ NK cells as percentage of human blood CD56^dim^ and CD56^bright^ NK cells. Statistically significant differences between the numbers of TRAIL expressing CD56^dim^ and CD56^bright^ NK cells are depicted with an asterisk (*). All data represent means+SEM.

Flow cytometric examination of the cell surface expression of TRAIL on human peripheral blood NK cells revealed that ∼6.2% of all NK cells expressed TRAIL (Fig.3A, B). Stimulation with SGBS ACM did not significantly affect the TRAIL expression. Independently from cultivation conditions, CD56^bright^ NK cells expressed TRAIL six times more than CD56^dim^ (Fig.3A, C).

### NK Cell Ability to Form Conjugates with Target Cells is not Altered After ACM Stimulation

**Figure 4:**
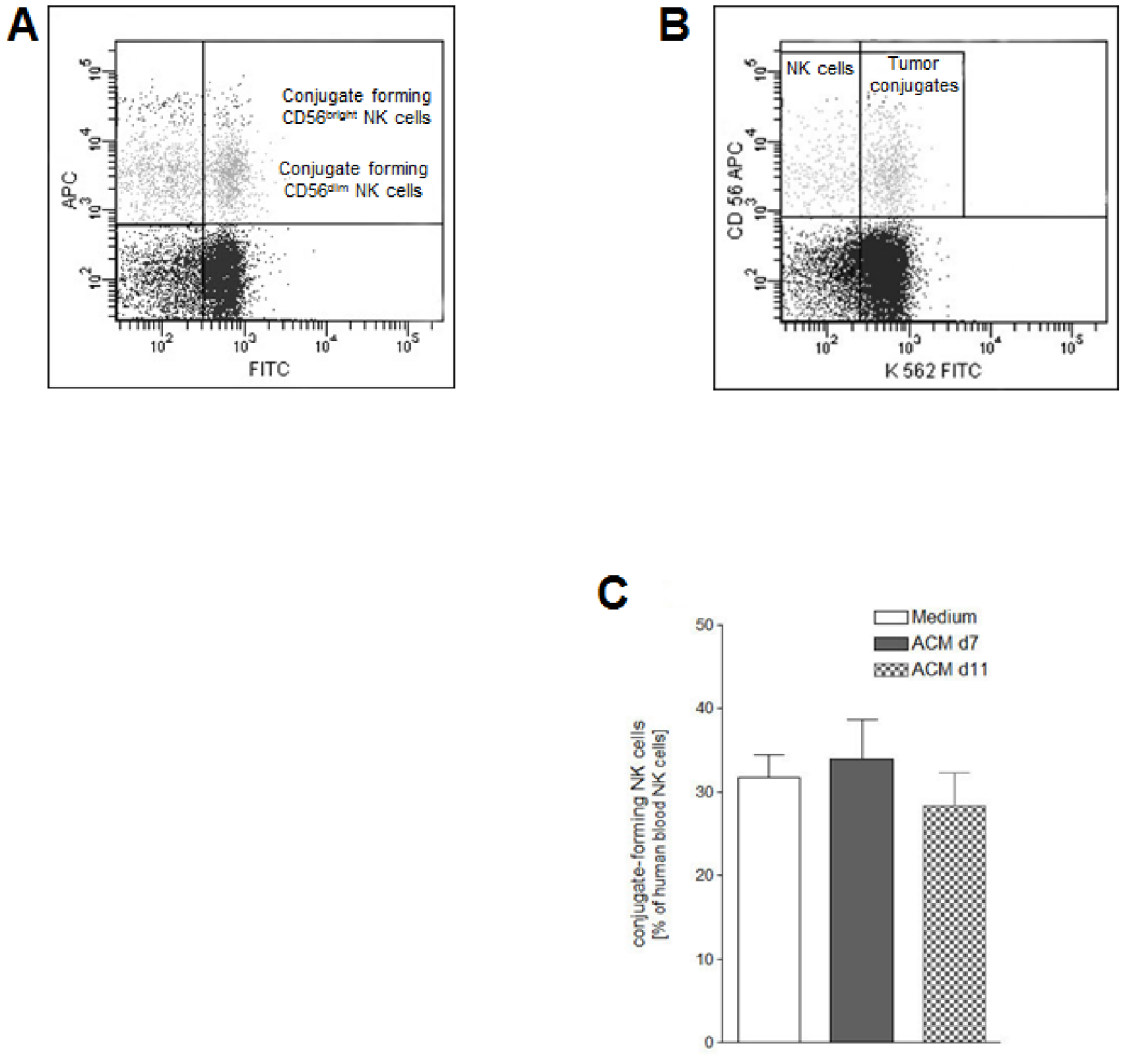
Ability of human blood NK cells to form conjugates with cells of the K562 erythroleukemia line after ACM stimulation. Human peripheral blood mononuclear cells (PBMCs) isolated from six leukocyte filters in each case were cultured for 24 hours with R10-medium conditioned with 1% of SGBS ACM harvested on day 7 or day 11 after induction of adipogenic differentiation. PBMCs incubated for 24 hours with medium only served as controls. A detailed description of the incubation procedure can be found in the materials and methods section. A) About 30% of the NK cells (CD3^−^CD56^+^) were able to form conjugates with K562 target cells (upper right quadrant) irrespective of the cultivation conditions. B) Conjugate forming human blood NK cells as percentage of human blood NK cells. All data represent means+SEM.

In order to study the direct interaction between NK cells and NK-sensitive target cells after 24 hours of SGBS ACM stimulation, PBMC stimulation was followed by incubation with cells from the erythroleukemia line K562 for 20 minutes. The subsequent flow cytometric analysis revealed that ∼30% of the NK cells were able to form conjugates with K562 cells (Fig. 4). SGBS ACM did not significantly alter the conjugate-forming ability of NK cells. We also found no difference between CD56^dim^ and CD56^bright^ NK cell populations concerning their ability to form conjugates with K562 cells.

### ACM Stimulation Distinctly Influences the Expression of Intracellular Cytokines in NK Cell Subsets

**Figure 5:**
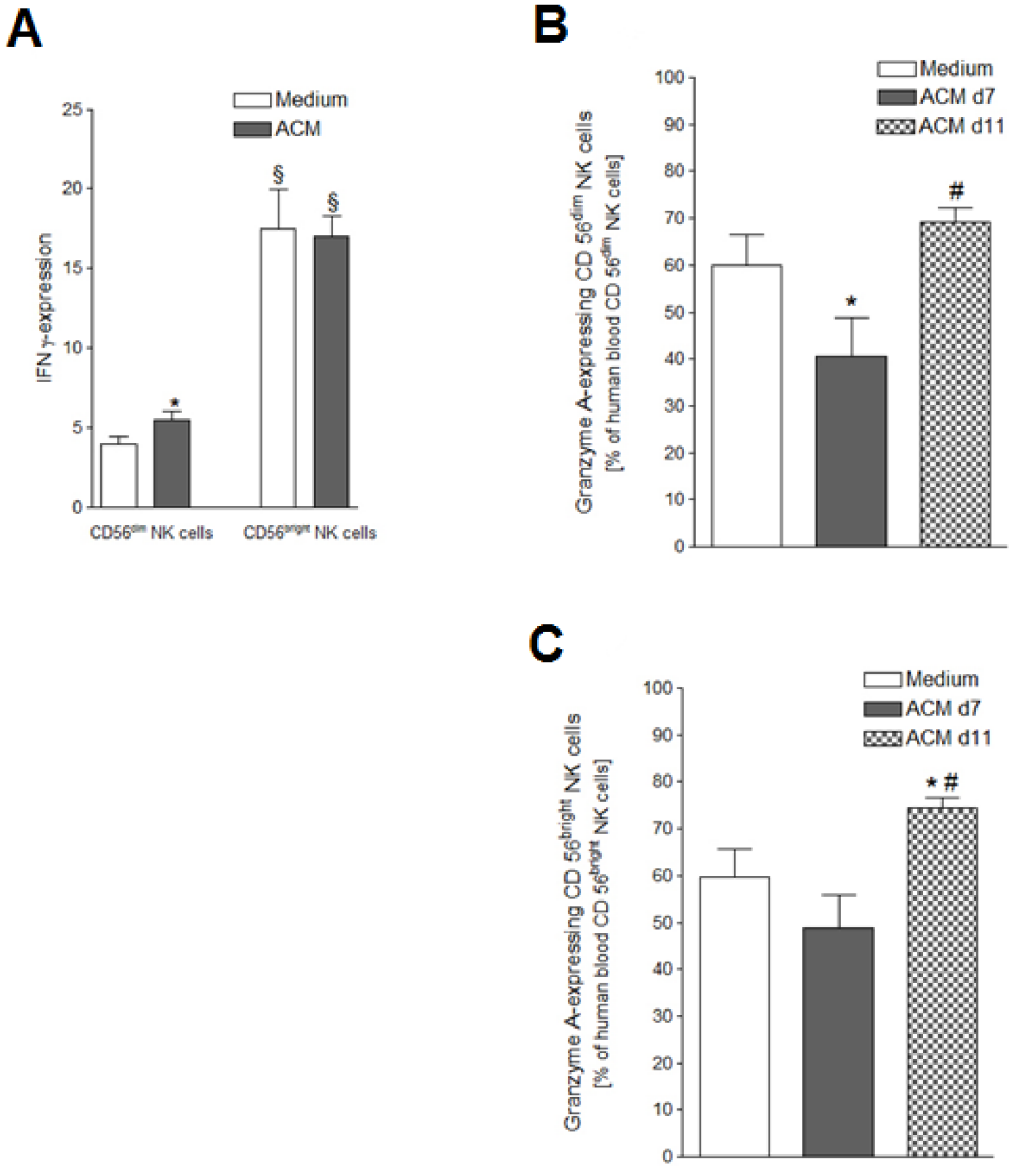
IFN-γ and GzmA expression after ACM stimulation. Human peripheral blood mononuclear cells (PBMCs) isolated from six leukocyte filters in each case were cultured for 24 hours with R10-medium conditioned with 1% of SGBS ACM harvested on day 7 (d7) or day 11 (d11) after induction of adipogenesis. PBMCs incubated for 24 hours with medium only served as controls. A detailed description of the incubation procedure can be found in the materials and methods section. A) IFN-γ-expressing human blood CD56^dim^ and CD56^bright^ NK cells as percentage of human blood CD56^dim^ and CD56^bright^ NK cells treated with 1% of the ACM harvested on day 7 after induction of adipogenic differentiation; B) GzmA-expressing human blood CD56^dim^ NK cells as percentage of human blood CD56^dim^ NK cells treated with 1% of the ACM harvested on day 7 or 11 after induction of adipogenic differentiation; C) GzmA-expressing human blood CD56^bright^ NK cells as percentage of human blood CD56^bright^ NK cells treated with 1% of the ACM harvested on day 7 or 11 after induction of adipogenic differentiation; Statistically significant differences between the stimulation with SGBS ACM and the medium control are depicted with an asterisk (*). Statistically significant differences between the stimulation with SGBS ACM harvested on day 7 after induction of adipocyte differentiation and the same dosage of SGBS ACM harvested on day 11 after induction of adipogenic differentiation are depicted with a rhomb (#). Statistically significant differences between the numbers of IFN-γ expressing CD56^dim^ and CD56^bright^ NK cells are depicted with a paragraph sign (§). All data represent means+SEM.

FACS analyses of the effect of a 24 hours SGBS ACM stimulation on the expression of intracellular IFN-γ by NK cells revealed that the CD56^bright^ NK cells showed a threefold higher IFN-γ expression as compared to the CD56^dim^ NK cell subset (Fig.5A). Additionally, the ACM stimulation lead to an increase in the IFN-γ expressing cells in the CD56^dim^ NK cell subset, whereas the already high IFN-γ expression level of the CD56^bright^ NK cells remained unchanged.

**Figure 6:**
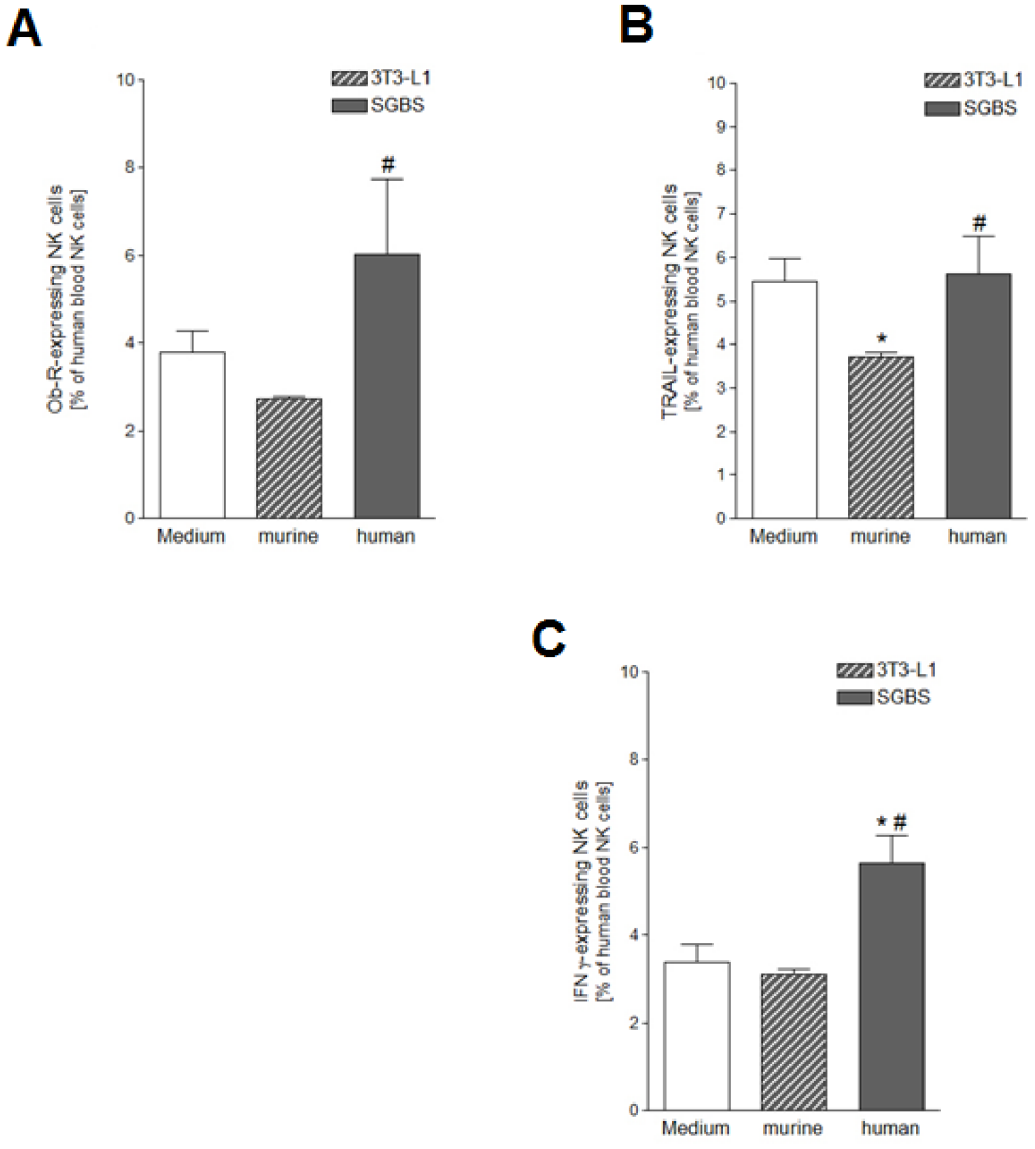
Ob-R, TRAIL and IFN-γ expression after stimulation with either murine or human ACM. PBMCs were cultured for 24 hours with R10-medium conditioned with 1% of either murine 3T3-L1 or human SGBS ACM harvested on day 7 after induction of adipocyte differentiation. PBMCs incubated for 24 hours with medium only served as controls. A detailed description of the incubation procedure can be found in the materials and methods section. A) Ob-R-expressing human blood NK cells as percentage of human blood NK cells; B) TRAIL-expressing human blood NK cells as percentage of human blood NK cells; C) IFN-γ-expressing human blood NK cells as percentage of human blood NK cells. Statistically significant differences between the stimulation with 3T3-L1 or SGBS ACM and the medium control are depicted with an asterisk (*). Statistically significant differences between the stimulation with SGBS ACM and 3T3-L1 ACM are depicted with a rhomb (#). All data represent means+SEM.

A difference in the stimulatory effects of the two ACM harvested on different days was seen regarding GzmA production by NK cells. The numbers of both NK cell subsets significantly increased after treatment with the ACM harvested on day 11 versus treatment with the day 7 ACM (Fig.6B+C). Culturing of the PBMCs with 1% of the day 7 ACM led to a significant decrease of the GzmA-expressing CD56^dim^ NK cells (Fig.5B). In contrast, after stimulation with the day 11 ACM an increase of GzmA-positive CD56^bright^ NK cells could be observed (Fig.6C).

### ACM-induced Effects on the Expression of Ob-R, IFN-γ and TRAIL in NK Cells are Species-specific

The human SGBS adipocyte strain was introduced ten years ago and has consecutively been shown to be comparable to primary human adipocytes in its gene expression profile during differentiation [24]. The murine 3T3-L1 cell line has been a well established model for the *in vitro* examination of adipogenesis and the secretory functions of adipocytes for a long time [25], [26]. Since species-specific differences between human and murine adipokines become more and more evident [27], we tested the species-specificity of our stimulation assay by repeating selected experiments with ACM of SGBS and 3T3-L1 adipocytes to compare the effects of human and murine ACM on NK cell functions. The ACM had both been collected on day 7 of adipogenesis, and in each case PBMCs isolated from six different leukocyte filters were cultured for 24 hours in R10-medium conditioned with either SGBS or 3T3-L1 ACM. FACS analyses were performed to determine the expression of Ob-R, TRAIL and IFN-γ expression.

After treatment with the 3T3-L1 ACM, numbers of NK cells expressing Ob-R or IFN-γ were not altered as compared to the medium control (Fig.6A, C). The levels of Ob-R^+^ NK cells were significantly higher after treatment with SGBS ACM as compared to the treatment with the murine 3T3-L1 ACM. While culturing of the PBMCs with the human ACM did not affect the TRAIL expression of NK cells, addition of the murine ACM led to a decrease in the number of TRAIL-positive NK cells (Fig.6B).

The number of IFN-γ-expressing NK cells was significantly higher after treatment with the SGBS ACM in comparison to the IFN-γ expression of cells treated with 3T3-L1 ACM or medium only (Fig.6C).

## Discussion

The present study was conducted to investigate the influence of an obese metabolic microenvironment on human peripheral blood NK cells. According to the density of their surface expression of CD56, human NK cells can be subdivided into two distinct subsets: CD56^dim^ and CD56^bright^ NK cells [28]. Our analyses showed that the intracellular cytokine production of NK cells and cell surface marker expression were influenced subset-specifically after stimulation with a heterogeneous cocktail of human adipokines.

The CD56^bright^ NK cell subset is assumed to act mainly via immunomodulatory actions, being the subset type which primarily secretes IFN-γ [28]. We observed that a significantly higher amount of the CD56^bright^ NK cells expressed intracellular IFN-γ as compared to the CD56^dim^ NK subset. Moreover, the proportion of cells expressing the death ligand TRAIL was 6-fold higher in the CD56^bright^ subset than in the CD56^dim^ NK cells. So far, little is known about differences between the two human NK cell subsets regarding their TRAIL expression pattern. TRAIL is constitutively expressed in human and murine liver NK cells [29], [30]. In humans, one report noted the majority of TRAIL to be expressed in the CD56^bright^ subset of NK cells in patients with chronic hepatits B [31]. As a subpopulation of murine NK cells was shown to express TRAIL as a result of the autocrine production of IFN-γ [30], [32], the greater occurrence of TRAIL^+^ cells in the CD56^bright^ compared to the CD56^dim^ NK cells could also be explained by a higher expression of IFN-γ in these cells. While the higher amount of IFN-γ-positive CD56^bright^ NK cells was independent from culturing conditions, the initially lower proportion of IFN-γ-expressing cells seen in the CD56^dim^ NK subset was increased after stimulation with ACM. In earlier investigations from our group a higher IFN-γ production of NK cells was seen after short-term (18 h) leptin treatment [33]. Long-term leptin stimulation, on the other hand, led to a decreased IFN-γ production by NK cells. Taking our present findings into consideration, it seems that this early increase in IFN-γ production is due to a higher IFN-γ expression by the CD56^dim^ NK cell subset. This is further supported by a report that observed a subset-specifically regulated IFN-γ production by NK cells upon receptor mediated stimulation. CD56^dim^ NK cells produced IFN-γ in the early phase of stimulation, while the long-term IFN-γ production is performed by the CD56^bright^ NK cell subset [34].

Adipose tissue is highly dynamic and adipocyte turnover occurs constantly in human adults with a replacement rate of about 10% of the fat cells per year [35]. The increase in adipose tissue mass during obesity results from both hypertrophic and hyperplastic growth [36]. It has been proposed to go along with adipose tissue dysfunction in terms of impaired adipogenesis and aberrant secretion patterns [37], [38], consequently resulting in changing circulating levels of adipokines [39].

The collection of ACM we utilized in our experiments was conducted on two different days of adipogenic differentiation. Thus they contain different concentrations and types of adipokines due to the respective secretory ability of SGBS adipocytes at the particular point of time during adipogenesis. SGBS adipocytes have been shown to be morphologically, biochemically and functionally identical to *in vitro* differentiated adipocytes from healthy human subjects. Their pattern and time-course of gene expression during differentiation was comparable to the findings in human preadipocytes in primary culture [24]. SGBS cells show a dynamic secretion pattern dependent of their state of differentiation [27], [39]. Though the majority of adipocyte secreted proteins (including leptin and adiponectin) is increasingly produced over the time span of adipocyte differentiation, the secretion levels of a minor fraction either decrease consistently or show a midterm peak level [24], [27]. Interestingly, the presence of the two ACM harvested on different days influenced the IFN-γ expression in NK cells similarly, whereas the expression of the cytotoxic enzyme GzmA was affected distinctly by each of the two different ACM. Stimulation with 1% of the ACM harvested on day 7 decreased the percentage of GzmA producing NK cells. Contrastingly, the same dosage of the day 11 ACM led to an increase in the GzmA expression in the CD56^bright^ NK cells without having this effect on the CD56^dim^ NK cells.

The most extensively studied adipokine is leptin and it affects immune cell functions via its receptor Ob-R [40] which is also expressed by human NK cells [41]. Our results show a similar distribution of Ob-R^+^ cells in both NK cell subsets with 4–8% of the cells expressing the leptin receptor. We observed slightly higher levels of Ob-R^+^ NK cells in overweight subjects (BMI ≥25 kg/m^2^) as compared to normal-weight subjects (BMI <25 kg/m^2^), consistent with a previous study that showed higher leptin receptor mRNA levels in DIO rats in comparison to lean littermates [19]. After an intravenous leptin injection, the Ob-Rb expression in NK cells from obese rats was up-regulated. This occurred together with a decreased NK cell activity and a lower activation of Ob-Rb postreceptor signaling components, suggesting a functional desensitization of leptin signaling in NK cells of obese animals. In a more recent study by our group an up-regulated Ob-R expression after leptin stimulation was also seen in human NK cells [33].We further noted a trend of higher peripheral blood NK cell numbers in the overweight group as compared to the normal-weight group. Our group previously showed higher NK cell numbers in DIO rats after adoptive transfer from obese rats as compared to lean recipients [18].

Studies concerning adipocyte differentiation and adipokine secretion have frequently been conducted with the well-established murine adipocyte cell line 3T3-L1 [25], [26]. However, the extrapolation of findings from these experiments to the human system is limited by the fact that adipokines show species-specific differences [27], [39]. The poor comparability of findings in humans and mouse models was recently confirmed by a study investigating the effects of inflammation on white blood cell gene expression [42]. Our studies showed distinct effects of human SGBS and murine 3T3-L1 ACM on NK cells. Thus, we suggest that the species-specificity of adipokine-immune interactions should also be considered in future studies.

Summing up, our results showed that human NK cell functions were altered after a 24 hours stimulation with human ACM. Our findings are in line with several studies reporting altered NK cell functions in obesity or after treatment with the adipokines leptin or adiponectin [18], [19], [22], [41], [43], [44]. Here, we could show that the in vitro exposition of PBMCs to an obese metabolic milieu differentially influenced the expression of intracellular IFN-γ and GzmA in the two distinct human NK cell subsets. Both cytokines, IFN-γ and GzmA, have been associated with autoinflammatory diseases. The relation between altered adipokine levels during obesity and associated autoimmune proinflammatory conditions, as seen in type 2 diabetes, is subject to extensive research. Our study gives further reference to the mechanisms by which human NK cell subsets may contribute to the chronic low-grade inflammatory state observed during obesity.
